# Activation of *PsMYB10.2* Transcription Causes Anthocyanin Accumulation in Flesh of the Red-Fleshed Mutant of ‘Sanyueli’ (*Prunus salicina* Lindl.)

**DOI:** 10.3389/fpls.2021.680469

**Published:** 2021-06-22

**Authors:** Zhi-Zhen Fang, Kui Lin-Wang, Dan-Rong Zhou, Yan-Juan Lin, Cui-Cui Jiang, Shao-Lin Pan, Richard V. Espley, Christelle M. Andre, Xin-Fu Ye

**Affiliations:** ^1^Fruit Research Institute, Fujian Academy of Agricultural Sciences, Fuzhou, China; ^2^Fujian Engineering and Technology Research Center for Deciduous Fruit Trees, Fujian Academy of Agricultural Sciences, Fuzhou, China; ^3^The New Zealand Institute for Plant and Food Research Limited, Mt Albert Research Centre, Auckland, New Zealand

**Keywords:** transcriptome, red-fleshed mutant, anthocyanin biosynthesis, *PsMYB10.2*, plum

## Abstract

Plum is one of the most important stone fruits in the world and anthocyanin-rich plums are increasingly popular due to their health-promoting potential. In this study, we investigated the mechanisms of anthocyanin accumulation in the flesh of the red-fleshed mutant of the yellow-fleshed plum ‘Sanyueli’. RNA-Seq and qRT-PCR showed that anthocyanin biosynthetic genes and the transcription factor *PsMYB10.2* were upregulated in the flesh of the mutant. Functional testing in tobacco leaves indicated that *PsMYB10.2* was an anthocyanin pathway activator and can activate the promoter of the anthocyanin biosynthetic genes *PsUFGT* and *PsGST*. The role of *PsMYB10.2* in anthocyanin accumulation in the flesh of plum was further confirmed by virus-induced gene silencing. These results provide information for further elucidating the underlying mechanisms of anthocyanin accumulation in the flesh of plum and for the breeding of new red-fleshed plum cultivars.

## Introduction

Plum is one of the most consumed stone fruits in the world and is a rich source of health-promoting compounds such as anthocyanins (Lee et al., 2009; Johnson et al., 2011; Valero et al., 2013; Fanning et al., 2014; Wang et al., 2020). Anthocyanins are responsible for the red pigmentation in plum skin and in the flesh of red-fleshed varieties (Cheng et al., 2016). It has been widely reported that the consumption of anthocyanin-rich foods promotes health (Santhakumar et al., 2015; Igwe et al., 2017; Jamar et al., 2017; Bakuradze et al., 2019). Increasing evidences have indicated that anthocyanins can delay the onset of diseases such as cancers, diabetes, cardiovascular and inflammatory diseases (He and Giusti, 2010). For example, Liu et al. (2017) reported that cyanidin, which is the major type of anthocyanin in plum fruits (Tomás-Barberán et al., 2001; Usenik et al., 2009; Wang R. et al., 2018), alleviates inflammation *in vivo* by blocking the binding of the cytokine interleukin-17A to the IL-17RA subunit, which suggested that cyanidin derived small molecules could be developed into drugs for the treatment of IL-17A–dependent inflammatory diseases and cancer.

China is the largest plum producer in the world (Milošević and Milošević, 2018), with a production >700 million tons in 2019^1^. While red-fleshed fruits are rich in anthocyanin and favored by consumers (Espley et al., 2013; Silvestri et al., 2018), they represent only one-fifth of the plum accessions in China (Zhang and Zhou, 1998). Most of the cultivated cultivars are red-skinned but yellow-fleshed. Therefore, it is of great interest to elucidate the mechanisms controlling anthocyanin accumulation in flesh of plum and develop new red-fleshed cultivars.

The anthocyanin biosynthesis pathway has been widely investigated in plants. The structural genes, such as *phenylalanine ammonia-lyase* (*PAL*), *cinnamate-4-hydroxylase* (*C4H*), *4-coumaroyl:CoA-ligase* (*4CL*), *chalcone synthase* (*CHS*), *chalcone isomerase* (*CHI*), *flavanone-3-hydroxylase* (*F3H*), *flavonoid3′-hydroxylase* (*F3′H*), *dihydroflavonol-4-reductase* (*DFR*), *anthocyanin synthase* (*ANS*), *UDPglucose:flavonoid-3-O-glucosyltransferase* (*UFGT*), and *glutathione S-transferase* (*GST*), that are involved in anthocyanin biosynthesis and accumulation are well documented (Tanaka et al., 2008; Jaakola, 2013). The expression of these genes are coordinately regulated by the MYB-bHLH-WD40 (MBW) complex (Allan et al., 2008; Rahim et al., 2014; Li et al., 2016) and other transcription factors, such as NACs, HD-Zips, bZIPs and WRKYs, which interact with the MBW complex or directly bind to the promoter of anthocyanin structural genes (Zhou H. et al., 2015; Jiang et al., 2017; An et al., 2018; Liu et al., 2018; Wang N. et al., 2018). Among them, the R2R3-MYBs have often been identified as the key positive or negative regulator (Espley et al., 2007; Feng et al., 2015; Tuan et al., 2015; Jin et al., 2016; Butelli et al., 2017; Plunkett et al., 2018; Zhang et al., 2018). In our previous study, we showed that a homolog of the *Arabidopsis R2R3 MYB* gene, *AtMYB113*, was upregulated during pigmentation of flesh in ‘Furongli’ plum (a red-fleshed cultivar) (Fang et al., 2016b). *R2R3-MYB* genes have been reported to be responsible for variation, loss or gain of anthocyanin pigmentation in several plants (Li et al., 2015; Kotepong et al., 2019; Wang et al., 2019; Jia et al., 2020; Xu et al., 2020). In addition, basic helix-loop-helix (bHLH) transcription factors also play an essential role in the regulation of anthocyanin biosynthesis in plant species such as Arabidopsis (Gonzalez et al., 2008), Petunia (Spelt et al., 2000), *Medicago truncatula* (Li et al., 2016), and grape (Hichri et al., 2010). Recently, it has been shown that abnormal *MabHLH3* expression is responsible for loss of anthocyanin accumulation in fruits of ‘Baiyuwang’ mulberry (Li et al., 2020). It has been demonstrated that bHLHs are indispensable for the anthocyanin activating activity in *Nicotiana* of peach PpMYB10.2 (Zhou et al., 2018) and apple MdMYB10 (Espley et al., 2007) and significantly enhanced the anthocyanin activating activity of peach PpMYB10.1 (Zhou H. et al., 2015) and PpMYB10.4 (Zhou et al., 2014) and Chinese bayberry MrMYB1 (Niu et al., 2010). bHLH proteins are characterized by a conserved bHLH domain, which is responsible for DNA recognition and DNA-binding specificity (Feller et al., 2011; Hichri et al., 2011). Zhou et al. (2018) showed that substitution of Arg/Gly^93^ in bHLH-binding domain of peach PpMYB10.1 and PpMYB10.2 could significantly affect their anthocyanin-promoting activity by altering their binding affinity to PpbHLH3.

Previously, we identified a red-fleshed and late-ripening mutant (named ‘Fuhongli’) from the radiation-induced mutant population obtained from ^60^Co-γ ray treated buds of ‘Sanyueli’ plum (Fang et al., 2016a). Due to its largely similar genetic origins, the mutant provides opportunities to investigate the molecular mechanisms that modulate anthocyanin accumulation in flesh of plum. This study aims to clarify the mechanisms underlying the accumulation of anthocyanins in the flesh of ‘Fuhongli’. The results indicated that anthocyanin content in the flesh of ‘Fuhongli’ increased rapidly during fruit ripening, but no anthocyanin was accumulated in the flesh of ‘Sanyueli’. RNA-Seq, dual-luciferase assays and transient color assays in tobacco leaves and plum fruits identified *PsMYB10.2*, which activated the promoters of *PsUFGT* and *PsGST*, as a key transcription factor contributing to anthocyanin accumulation in the flesh of ‘Fuhongli’. The present study suggests that *PsMYB10.2* is a weak anthocyanin activator and is responsible for anthocyanin biosynthesis in flesh of plum fruits.

## Materials and Methods

### Plant Materials

All fruits were harvested from 5-years old ‘Sanyueli’ (SY) plum (*Prunus salicina* Lindl.) tree and its red-fleshed mutant ‘Fuhongli’ (MT) grown in Fruit Research Institute, Fujian Academy of Agricultural Sciences, Fuzhou, Fujian Province, China. Fruit samples of ‘Sanyueli’ were collected at 95, 105, 115 days after flowering (DAF) and Fruit samples of red-fleshed mutant were collected at 95, 105, 115, and 125 DAF. Fruit maturity in ‘Sanyueli’ plums was reached 115 days after flowering (DAF), while ‘Fuhongli’ plums ripened 150 DAF. Fruits sampled from three different branches were used as biological replicates. Ten representative fruits sampled from each branch at each developmental stage were peeled and sliced into small pieces. The sliced fruit fleshes were pooled into three replicates and immediately frozen in liquid nitrogen and kept at −80°C for further experiments.

### Determination of Total Sugar, Fructose, Glucose, Sucrose, and Organic Acid Contents

Concentrations of total sugar, fructose, glucose and sucrose were measured using total sugar (ZT-1-Y), fructose (GT-1-Y), glucose (PT-1-Y), sucrose (ZHT-1-Y) assay kits (Comin Biotechnology Co., Ltd., Suzhou, China) according to the manufacturer’s instructions. Titratable acid was measured as described by Chen and Zhu (2011). For the determination of malic acid content, 0.1 g powder was suspended with 1 ml ice-cold water and extracted by ultrasonic treatment for 1 h. The extracts were centrifugated at 8,000 × g for 10 min. The supernatant was filtrated ussing a 0.22 μm membrane filter and then analyzed with HPLC using an Agilent 1100 system (Agilent Technologies, Palo Alto, CA, United States). Separation was performed using a Kromasil C18 250 × 4.6mm, 5μm column (AkzoNobel, Sweden).

### Determination of Anthocyanin and Carotenoid Content in Plum Flesh

For quantification of anthocyanin, approximately 2 g of fruit flesh was ground to fine powder in liquid nitrogen and extracted in 10 mL methanol with 0.05% HCl at 4°C for 24 h. Anthocyanin content was quantified by pH differential method as described previously (Fang et al., 2016b). Three measurements for each biological replicate sample were performed. The total carotenoid content was measured using the carotenoid assay kit (Comin Biotechnology Co., Ltd., Suzhou, China.) as previously described (Hou et al., 2020).

For HPLC analysis, approximately 2 g of the flesh of mature fruits of ‘Fuhongli’ was ground in liquid nitrogen and extracted in 20 ml methanol with 1% HCl at 4°C for 12 h in the dark. Samples were brought to room temperature then centrifuged for 10 min at 5,866 × g. HPLC analysis was performed using an Agilent 1120 system (Agilent Technologies, Palo Alto, CA). Separation was performed using a C-18 4.6 × 150 mm column (InfinityLab Poroshell 120 EC-C18, Agilent, United States). The mobile phase was 0.3% phosphoric acid in water (solvent A) and acetonitrile (solvent B) at a flow rate of 1 ml min^–1^. The linear gradient of phase B was as follows: 0–20 min, 5%; 20–30 min, 15%; 30–40 min, 25%; 40–40 min, 25%; 48–50 min, 5%. The injection volume was 10 μL. Anthocyanin components were detected at 510 nm and peaks identified by comparison of peaks to authentic standards.

### RNA Preparation, Library Construction and RNA-Seq

Extraction of total RNA, library construction and RNA-Seq were performed by staff at Beijing BioMarker Technologies (Beijing, China) as described previously (Fang et al., 2016b). Sequencing of the cDNA library was carried out on the Illumina HiSeq^TM^ X Ten sequencing platform. RNA-seq was performed in three replicates. The RNA-seq reads have been deposited in the NCBI Short Read Archive and are accessible under PRJNA690967.

### Analysis of RNA-Seq Data

The RNA-seq raw reads were processed with in-house perl scripts to remove reads containing adapter, reads containing ploy-N and low-quality reads. All generated clean reads were mapped to the genome sequence of ‘Sanyueli’ (Fang et al., 2020) using HISAT2 (Kim et al., 2015). The expression level of genes was estimated by the fragments per kilobase of transcript per million fragments mapped (FPKM) values using the StringTie software package (Pertea et al., 2015). DESeq2 (Love et al., 2014) was employed to perform differential gene expression analysis. Genes with fold change ≥2 and a false discovery rate <0.01 were considered significantly differentially expressed. One sample of ‘Sanyueli’ at 105DAF (SY105d-2) which was not well correlated with other biological replicates at the same stage was excluded from further analysis. GO and KEGG pathway enrichment analysis was carried out as reported previously (Fang et al., 2020). The heat maps for differentially expressed genes were constructed using TBtools (Chen et al., 2020).

### Prediction of Transcription Factors

All transcripts were subjected to the PlantTFDB v5.0^2^ to predict transcription factors using the Transcription Factor Prediction module.

### Real-Time Quantitative RT-PCR (qRT-PCR) Analysis

The expression of 15 differentially expressed genes were validated using qRT-PCR. The extraction of total RNA from fruit flesh was carried out using a modified CTAB method (Xuan et al., 2015). All primers used for qRT-PCR were provided in Supplementary Table 1. The synthesis of cDNA was carried out using the PrimeScript^®^ RT reagent kit with gDNA Eraser (Takara, Dalian, China). qRT-PCR was performed using the Eppendorf Realplex^4^ real-time PCR system (Hamburg, Germany) as described previously (Fang et al., 2016b). qRT-PCR conditions were 5 min at 95°C, followed by 40 cycles of 5 s at 95°C, 15 s at 60°C, and 30 s at 72°C, followed by 60–95°C melting curve detection. *Actin* (PsSY0026636) gene was used as the reference. The expression levels were calculated as described previously (Fang et al., 2016b). Three biological and three technical replications were performed.

### Phylogenetic Analysis and Multiple Sequence Alignment

Phylogenetic and evolutionary analyses were performed using the neighbor-joining method with 1000 bootstrap replicates by MEGA-X. Additional sequences include *Arabidopsis thaliana* AtMYB4 (At4G38620), AtMYB11 (AT3G62610), AtMYB12 (AT2G47460), AtMYB75 (AT1G56650), AtMYB90(AT1G66390), AtMYB111 (AT5G49330), AtMYB113 (AT1G66370), AtMYB114 (AT1G66380), AtMYB123 (AT5G35550); *Actinidia chinensis* AcMYB10 (PSS35990) and AcMYB110 (AHY00342); *Fragaria* × *ananassa* FaMYB1 (AAK84064.1), FaMYB9(JQ989281), FaMYB10 (ABX79947.1); *Gossypium hirsutum* GhMYB36(AF336284); *Lotus japonicus* LjMYB12 (AB334529); *Myrciaria cauliflora* McMYB (MH383068); *Malus* × *domestica* MdMYB9 (DQ267900), MdMYB10 (EU518249), MdMYB11 (DQ074463); *Medicago truncatula* MtLAP1 (ACN79541.1) and MtMYB14 (XP_003594801.1); *Prunus cerasifera* PcMYB10.1 (KP772281) and PcMYB10.2 (KP772282); *Petunia hybrida* PhMYB27 (AHX24372), PhAN2 (AB982128), PhDEEP PURPLE (ADQ00393.1), PhPURPLE HAZE (ADQ00388.1), and PhMYB4 (ADX33331.1); *Prunus persica* PpMYB10.1(XM_007216468), PpMYB10.2(XM_007216161), and PpMYB18 (KT159234); *Prunus salicina* PsMYB10.1(MK105923) and PsMYB18 (MK284223); *Pyrus pyrifolia* PyMYB10(GU253310); *Solanum lycopersicum* SlMYB12 (ACB46530.1); *Trifolium arvense TaMYB14* (AFJ53053.1); *Trifolium repens* TrMYB4 AMB27079), TrMYB133 (AMB27081) and TrMYB134 (AMB27082); *Vaccinium corymbosum* VcMYBA (MH105054); *Vaccinium uliginosum* VuMYBC2; *Vitis vinifera* VvMYBA1 (BAD18977), VvMYBA2(BAD18978), VvMYBF1 (ACV81697), VvMYBC2-L1 (AFX64995.1), VvMYB-L2 (ACX50288.2), VvMYBPA1 (CAJ90831.1), and VvMYBPA2 (ACK56131.1). Multiple sequence alignment of R2R3 MYB amino acid sequences from plum, sweet cherry and peach was performed by DANMAN7.

### Vector Construction

cDNA was generated from red pigmented flesh of ‘Fuhongli’ using a RevertAid First-Strand cDNA synthesis kit (Thermo Fisher Scientific, Waltham, MA). *PsMYB10.2* cDNA and *PsbHLH3* cDNA were isolated using 2 × Phanta Max Master Mix (Vazyme, Nanjing, China) and inserted into pSAK277. Genomic DNA was isolated from young plum leaves using a Plant Genomic DNA kit (Tiangen, China) and used as a template for promoter amplification. Promoter sequences of *PsUFGT* (2523bp) and *PsGST* (2339bp) were amplified and inserted into pGreenII 0800–LUC vector using ClonExpress Ultra One Step Cloning Kit (Vazyme, Nanjing, China). A fragment of *PsMYB10.2* (296–645 bp) was amplified using specific primers (TRV2-PsMYB10.2F and pTRV2-PsMYB10.2R) and inserted into pTRV2 to generate pTRV2-PsMYB10.2. Primer sequences used for the vector construction are listed in Supplementary Table 2.

### Transient Color Assay in Tobacco Leaf and Quantification of Anthocyanins

Transient overexpression assays were performed in young leaves of 2-week-old seedlings of *Nicotiana tabacum* grown in the greenhouse as reported by Zhou et al. (2019) using *Agrobacterium tumefaciens* strain GV3101. *A. tumefaciens* GV3101 carrying constructs were incubated at 28°C for 2 days and resuspended in infiltration buffer containing 10 mM MgCl_2_, 100 μM acetosyringone (pH = 5.7) to an OD_600_ of 0.5. Separate strains containing *PsMYB10.2* and *PsbHLH3* fused to the 35S promoter in the pSAK277 vector and empty pSAK277 vector were infiltrated or co-infiltrated into the abaxial leaf surface. Peach *PpMYB10.1* (Zhou H. et al., 2015) co-infiltrated with *PsbHLH3* was used as a positive control. Photographs were taken 7 days after infiltration.

For quantification of anthocyanins, 10 mg of freeze-dried tobacco leaves from the infiltrated area was mixed in 1 mL of methanol: water: formic acid (80: 19.5: 0.5, *v/v/v*) and shaken for 4 h at room temperature. The tube containing the mixture was centrifuged at 10,000 g for 15 min. The supernatants were filtered through a 0.45 μm PTFE syringe filter and submitted for high performance liquid chromatography (HPLC) analysis according to a method reported by Andre et al. (2012) with a few modifications. Briefly, quantification of the anthocyanins was performed using a Dionex Ultimate 3000 system (Sunnyvale, CA) equipped with a diode array detector (DAD). A 5 μL aliquot was injected onto a Thermo C18 Acclaim PolarAdvantage II column (150 × 2.1 mm i.d.; 3 μm particle size) (Waltham, MA). The mobile phases were (A) H_2_O with 5% formic acid and (B) MeCN with 0.1% formic acid. The flow rate was 0.35 mL min^–1^, and the column temperature was 35°C. The 28 min gradient was as follows: 0-5 min, 7% B constant; 5-10 min, 7-12% B; 10-20 min, 12-25% B; 20-21 min, 25-100% B; 21-24 min, 100% B constant; 24 min, 7% B; 24-28 min, 7% B re-equilibration time. Monitoring was set at 520 nm for quantification. Anthocyanins were identified by their spectral data and were quantified as cyanidin-3-glucoside using five-point calibration curves. A validation standard was injected after every 10th injection.

### Dual-Luciferase Assay

Dual-luciferase assays were conducted in *N. benthamiana* leaves as reported previously (Lin-Wang et al., 2010). Promoter constructs were individually transformed into *A. tumefaciens* GV3101 with the pSoup plasmid. Agrobacteria cultivation was performed as described above for transient color assay. For dual-luciferase assays, Agrobacteria strain GV3101 were transformed with the constructs and incubated at 28°C for 2 days and resuspended in infiltration buffer containing 10 mM MgCl2, 100 μM acetosyringone (pH = 5.7) to an OD_600_ of 0.5. Two *Agrobacteria* suspensions carrying the overexpression vectors (empty vector, *PsMYB10.2* and *PsbHLH3*) were mixed equally and then combined with one of the dual-luciferase promoter construct (*pPsUFGT:LUC* and *pPsGST: LUC*) suspensions in a 5:1 ratio. The mixture of *Agrobacteria* suspension carrying an empty vector control construct and the dual-luciferase promoter construct, again suspensded in a 5:1 ratio, was infiltrated as control. The final mixture was infiltrated into the abaxial leaf surface of *N. benthamiana* using a needleless 1 ml syringe. Four days after infiltration, 3-mm diameter leaf punches from infiltrated patches were harvested and subjected to dual-luciferase assay.

### RNAi Transient Assay of Plum Fruit Using the Virus-Induced Gene Silencing (VIGS) System

pTRV1, pTRV2, and pTRV2-PsMYB10.2 were individually transformed into *Agrobacterium* strain GV3101. These *Agrobacterium* strains were suspended in infiltration buffer (10 mM MgCl_2_, 10 mM MES, pH 5.6, 150 μM acetosyringone) and kept at room temperature for 3 h. Fruit infiltration was performed using maturing fruit of ‘Fuhongli’ as previously described (Zhou H. et al., 2015). Flesh tissues around the infiltration site were harvested and used for quantification of anthocyanin concentration and qRT-PCR analysis. Quantification of anthocyanin content was performed as described previously (Fang et al., 2016b).

## Results

### Accumulation of Anthocyanin in ‘Fuhongli’

**FIGURE 1:**
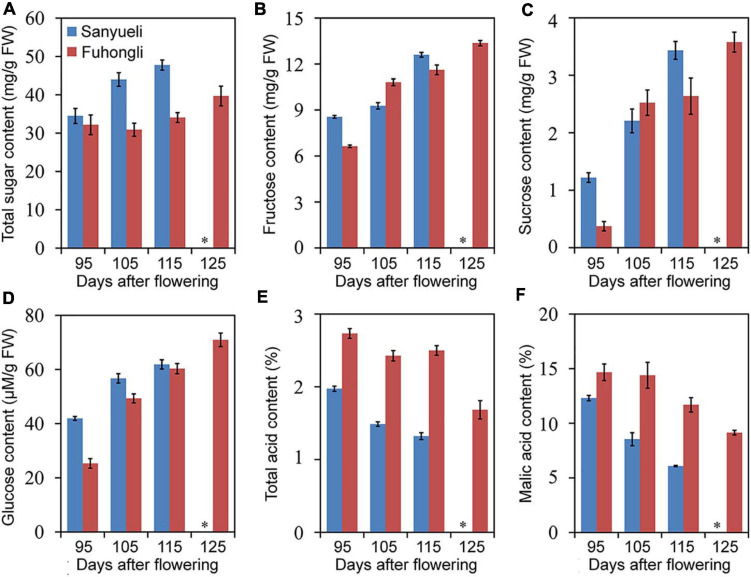
Trends in total soluble sugar **(A)**, fructose **(B)**, sucrose **(C)**, glucose **(D)**, total acid **(E)**, and malic acid **(F)** content in the flesh of ‘Sanyueli’ and ‘Fuhongli’ plum during fruit ripening. ^∗^ indicates there is no value for ‘Sanyueli’ 125 DAF as the fruits ripened earlier. Error bars represent standard error of three replicates.

**FIGURE 2:**
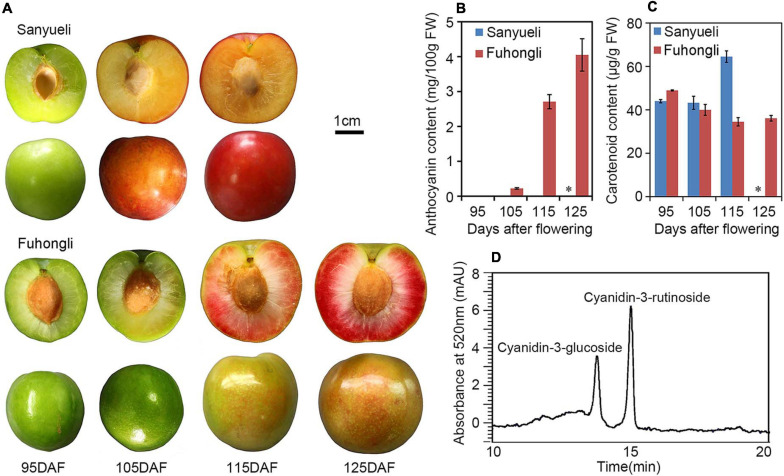
Pigmentation changes during fruit ripening of ‘Sanyueli’ and ‘Fuhongli’ plum. **(A)** Fruits of ‘Sanyueli’ plum and ‘Fuhongli’ at different stages. **(B)** Comparison of anthocyanin content in different ripening stages of ‘Sanyueli’ and ‘Fuhongli’. **(C)** Carotenoid content in different ripening stages of ‘Sanyueli’ and ‘Fuhongli’. **(D)** HPLC chromatogram of anthocyanins extracted from flesh of ‘Fuhongli’. ^∗^ indicates there is no value for ‘Sanyueli’ 125 DAF as the fruits ripened earlier. Error bars represent standard error of three replicates.

The dynamic changes in content of total soluble sugar, fructose, sucrose, glucose, total acid and malic acid were analyzed in the ‘Sanyueli’ and ‘Fuhongli’ during ripening. The accumulation pattern of total soluble sugar, fructose, sucrose and glucose were similar in ‘Sanyueli’ and ‘Fuhongli’ (Figures 1A–D). The content of total acid and malic acid decreased during the stages of fruit ripening in both ‘Sanyueli’ and ‘Fuhongli’ (Figures 1E,F). The flesh of unripe ‘Sanyueli’ fruit was green and changed into yellow during the late stages of ripening (Figure 2A). The flesh color of ‘Fuhongli’ was similar to that of ‘Sanyueli’ during the early stages of fruit development. However, the flesh of ‘Fuhongli’ showed weak red pigmentation at 105DAF and rapidly turned red during the late stages of ripening (Figure 2A). Analysis of fruit flesh anthocyanin contents demonstrated that no anthocyanin was accumulated in the flesh of ‘Sanyueli’ (Figure 2B). In contrast, a small amount of anthocyanins was detected in the flesh of ‘Fuhongli’ at 105DAF, when the fruit skin was still green, and the content of anthocyanin increased significantly during the late stages of ripening (Figure 2B). The carotenoid content increased in the flesh of ‘Sanyueli’, but slightly decreased in the flesh of ‘Fuhongli’ (Figure 2C). According to HPLC analysis, the anthocyanins accumulated in the flesh of mature fruits of ‘Fuhongli’ were cyanidin-3-rutinoside and cyanidin-3-glucoside (Figure 2D).

### RNA-Seq and Identification of Differentially Expressed Genes(DEGs) During Fruit Ripening of ‘Sanyueli’ and ‘Fuhongli’

Flesh of ‘Sanyueli’ and ‘Fuhongli’ at different stages were subjected to RNA-seq analysis. A total of 163.32Gb clean data were obtained from these samples after discarding low-quality reads and adaptor sequences (Supplementary Table 3). The clean reads were mapped to the genome of ‘Sanyueli’ (Fang et al., 2020). The mapping ratio to the reference genome ranged between 81.29% and 85.35% (Supplementary Table 3). Correlation analysis showed that the biological replicates were highly correlated within each stage (Supplementary Figure 1). A total of 2,091 putative new genes that have not appeared in the genome of ‘Sanyueli’ were identified and 1,641 of them was annotated by databases including NCBI non-redundant protein database (NR), Swiss-Prot protein database (Swiss-Prot), Gene Ontology (GO), Clusters of Orthologous Groups database (COG), euKaryotic Ortholog Groups of proteins database (KOG), protein family database (Pfam) and Kyoto Encyclopedia of Genes and Genomes (KEGG) (Supplementary Table 4).

A total of 9,594 genes were identified to be differentially expressed by pairwise comparison (Supplementary Figure 2). Among them, 3,073 and 3,000 genes were differentially expressed during ripening of ‘Sanyueli’ and ‘Fuhongli’, respectively. The comparison of transcriptome between ‘Sanyueli’ and ‘Fuhongli’ at different stages enabled the identification of 8477 DEGs (Supplementary Figure 2).

### Identification of Differentially Expressed Structural Genes Involved in Anthocyanin Accumulation

**FIGURE 3:**
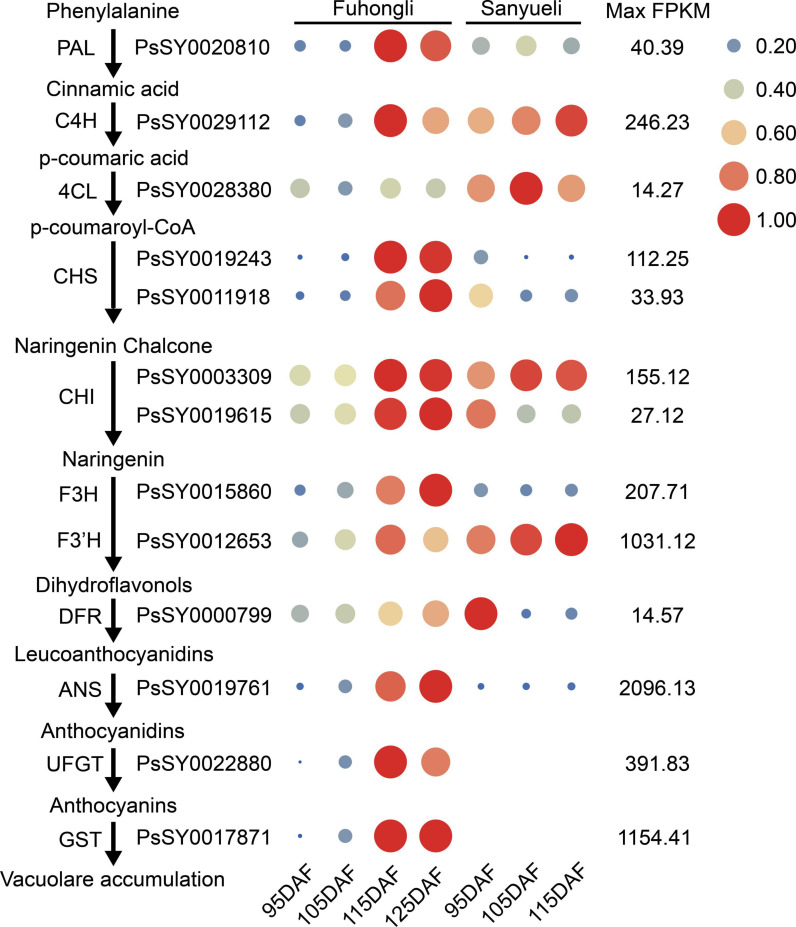
Expression of structural genes involved in the phenylpropanoid and anthocyanin biosynthesis, as evaluated by RNA-sequencing in the plum flesh of ‘Sanyueli’ and ‘Fuhongli’. FPKM values of all genes were normalized with maximum FPKM values of each gene. The expression level of genes was indicated using filled circle with different size and color. The larger the circle, the higher the expression. High expression was indicated in red, while low expression was indicated in blue. The values on the right indicates the highest FPKM value of each gene. Abbreviations for pathway genes as described in the Introduction.

**FIGURE 4:**
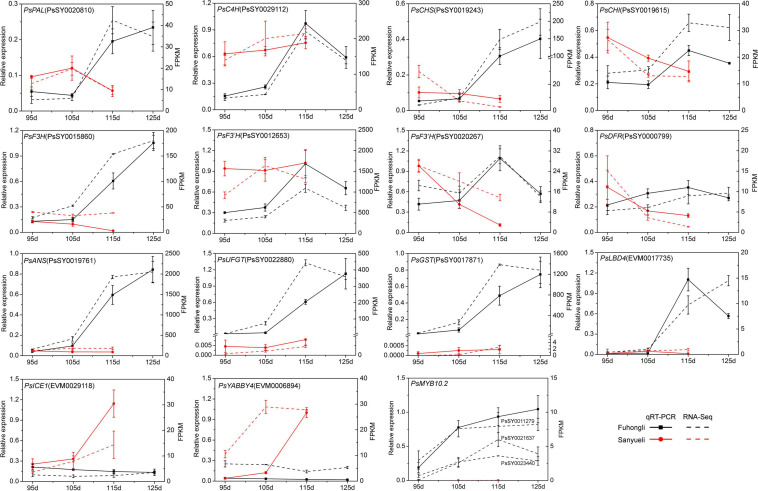
Expression profiles of selected differentially expressed transcription factors in the flesh of ‘Sanyueli’ and ‘Fuhongli’. FPKM values of all genes were normalized with maximum FPKM values of each gene. The expression level of genes was indicated using filled circle with different size and color. The larger the circle, the higher the expression. The values on the right indicates the highest FPKM value of each gene.

To investigate in which step of anthocyanin synthesis was activated in the red flesh of ‘Fuhongli’, we identified differentially expressed structural genes of anthocyanin biosynthesis according to the KEGG and GO annotation results. In total, 249 genes were assigned to phenylpropanoid biosynthesis and flavonoid biosynthesis pathway according to KEGG enrichment analysis (Supplementary Table 5). Ten of them, including *PAL* (PsSY0020810), *C4H* (PsSY0029112), *4CL* (PsSY0028380), *CHS* (PsSY0019243 and PsSY0011918), *CHI* (PsSY0003309), *F3H* (PsSY0015860), *F3′H* (PsSY0012653), *DFR* (PsSY0000799), and *ANS* (PsSY0019761), were identified as DEGs (Figure 3). PsSY0019615 was annotated as CHI and encodes a protein with molecular function designated as chalcone isomerase activity (GO:0045430) and involved in anthocyanin-containing compound biosynthetic process (GO:0009718). PsSY0020267 was annotated as *F3′H* and predicted to have flavonoid 3′,5′-hydroxylase activity (GO:0033772). PsSY0022880 was annotated as anthocyanidin 3-O-glucosyltransferase having anthocyanidin 3-O-glucosyltransferase activity (GO:0047213). In addition, PsSY0017871 was predicted to encode a GST which was shown to be upregulated in the flesh of red-fleshed cultivar ‘Furongli’ plum during fruit ripening in our previous study (Fang et al., 2016b). All of these anthocyanin-related genes, except *4CL*, were upregulated during fruit ripening of ‘Fuhongli’ (Figure 3 and Supplementary Table 5). There was no significant change in the expression of *PAL* (PsSY0020810), *C4H* (PsSY0029112), *CHS* (PsSY0003309), *F3H* (PsSY0015860), *F3′H* (PsSY0012653), and *ANS* (PsSY0019761) in the flesh of ‘Sanyueli’ during fruit ripening, while that of *CHS* (PsSY0019243 and PsSY0011918), *CHI* (PsSY0019615), and *DFR* (PsSY0000799) was significantly downregulated (Figure 3 and Supplementary Table 5). It is noteworthy that the expression of *C4H* (PsSY0029112), *CHS* (PsSY0003309) and *F3′H* (PsSY0012653) in the flesh of ‘Sanyueli’ was comparable to the highest level in the flesh of ‘Fuhongli’, but *UFGT* (PsSY0022880) and *GST* (PsSY0017871) were only expressed at extremely low levels in the flesh of ‘Sanyueli’ (Figure 3 and Supplementary Table 5). The expression profiles of 11 differentially expressed structural genes were validated using qRT-PCR. The results showed that the expression patterns detected by qRT-PCR experiments of the selected genes were consistent with the expression patterns investigated by RNA-seq analysis (Figure 4).

### Identification of Transcription Factors

**FIGURE 5:**
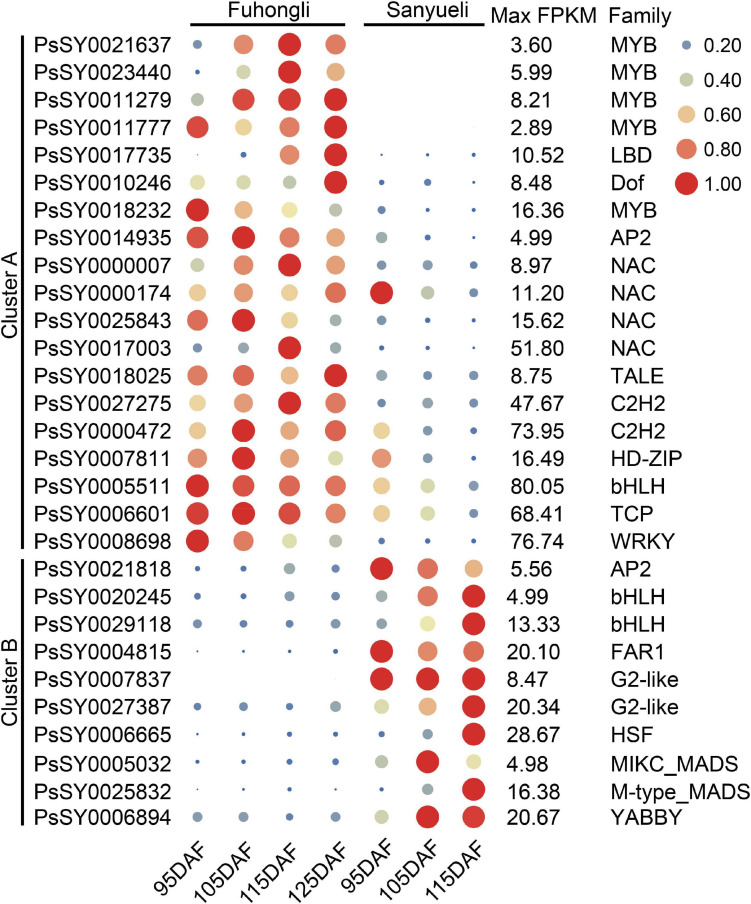
qRT-PCR validation of DEGs in flesh of ‘Sanyueli’ and ‘Fuhongli’ during fruit ripening. *Actin* was used as the reference gene. The error bars represent the standard error of three biological replicates. The left y-axis shows the relative gene expression levels obtained by qRT-PCR (solid lines). The right y-axis indicates the corresponding results of RNA-seq (dashed lines).

Since the structural genes involved in anthocyanin accumulation are transcriptionally regulated, we examined identify transcription factor potentially related to anthocyanin accumulation in the flesh of ‘Fuhongli’. From this, 1687 genes were predicted to encode transcription factors and 536 of them were differentially expressed (Supplementary Table 6). A total of 29 transcription factor genes, including 19 genes (Cluster A) that were increased in the flesh of ‘Fuhongli’ but expressed at low level or decreased in the flesh of ‘Sanyueli’ and 10 genes (Cluster B) that were expressed at low level in the flesh of ‘Fuhongli’ but increased or expressed in higher level in the flesh of ‘Sanyueli’, were identified (Figure 5 and Supplementary Table 6). These include five MYBs and the best hit in *Arabidopsis* to four of them is AtMYB113 (Figure 5 and Supplementary Table 6). PsSY0018232, which was decreased in both ‘Sanyueli’ and ‘Fuhongli’ but more abundant in ‘Fuhongli’ during fruit ripening, has high sequence similarity with MYB repressors such as PpMYB6. Intriguingly, the expression of a gene annotated as *LOB domain-containing protein 4* (*LBD4*, PsSY0017735) was significantly enhanced in the flesh of ‘Fuhongli’ during pigmentation but only expressed at a low level in the flesh of ‘Sanyueli’ (Figures 4, 5 and Supplementary Table 6). In addition, many other TF types including NACs, Dof, AP2, G2-like, FAR1, and MADS were also included in the differentially expressed transcription factor sets (Figure 5 and **Supplementary Table 6**). The expression pattern of *PsICE1* and *PsYABBY4*, which are lowly expressed in the flesh of ‘Sanyueli’, was also confirmed by qRT-PCR (Figure 4).

### *PsMYB10.2* Is a Positive Regulator of Anthocyanin Biosynthesis

**FIGURE 6:**
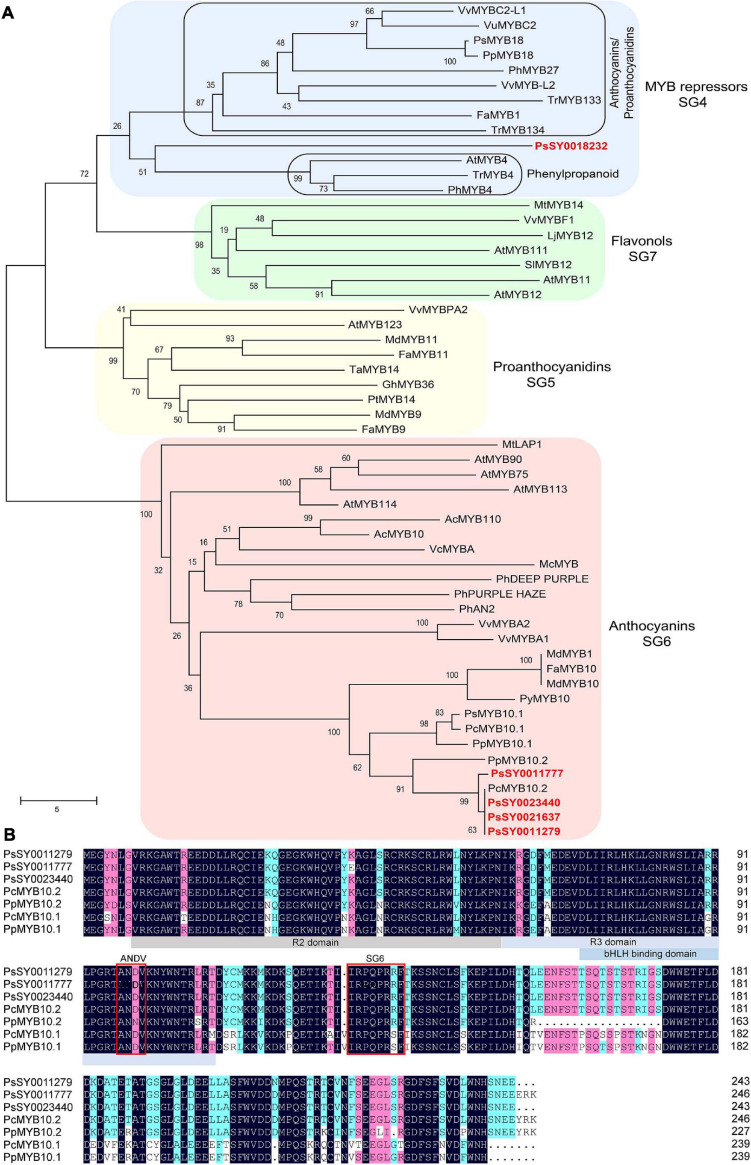
Sequence analysis of selected R2R3 MYBs. **(A)** Phylogenetic analysis of PsMYBs and R2R3 MYBs from other plant species. Plum MYBs are indicated in red font. **(B)** Multiple sequence alignment of PsMYBs and R2R3 MYBs from peach and cherry plum. R2, R3, and bHLH binding domain are highlighted in gray and blue colors, conserved ‘ANDV’ and SG6 motif for anthocyanin-promoting MYBs are indicated in red boxes.

Nucleotide sequence alignment results demonstrated that the coding region of the four *PsMYB* genes (PsSY0021637, PsSY0023440, PsSY0011279, and PsSY0011777) that were upregulated in the flesh of ‘Fuhongli’ were nearly identical (Supplementary Figure 3). The genomic DNA sequence of *PsMYB* genes was also nearly identical. However, a 7,045 nt insertion was detected in the second intron of PsSY0011777 (Supplementary Figure 4). The PsSY0021637, PsSY0023440, and PsSY0011279 encode a 732 bp coding sequence and predicted protein with 243 amino acid residues while PsSY0011777 encode a 741 bp coding sequence and predicted protein with 247 amino acid residues. Phylogenetic analysis showed that all of them fell in the anthocyanin clade (SG6) and were clustered into a subgroup with previously reported anthocyanin-activating MYB proteins from other Rosacea species (Figure 6A). The two proteins most closely related to the PsMYBs were PcMYB10.2 and PpMYB10.2 from cherry plum and peach (Figure 6A). Multiple sequence alignment showed the PsMYBs contain conserved R2R3 domain and ‘ANDV’ and SG6 motif for anthocyanin-promoting MYBs. They are highly homologous to PcMYB10.2 and PpMYB10.2, but PpMYB10.2 had an 18-amino acid deletion and PsMYBs (PsSY0021637 and PsSY0011279) had a 3-amino acid deletion in the C-terminal region (Figure 6B). These results suggest that the PsMYBs are likely activators of anthocyanin biosynthesis.

**FIGURE 7:**
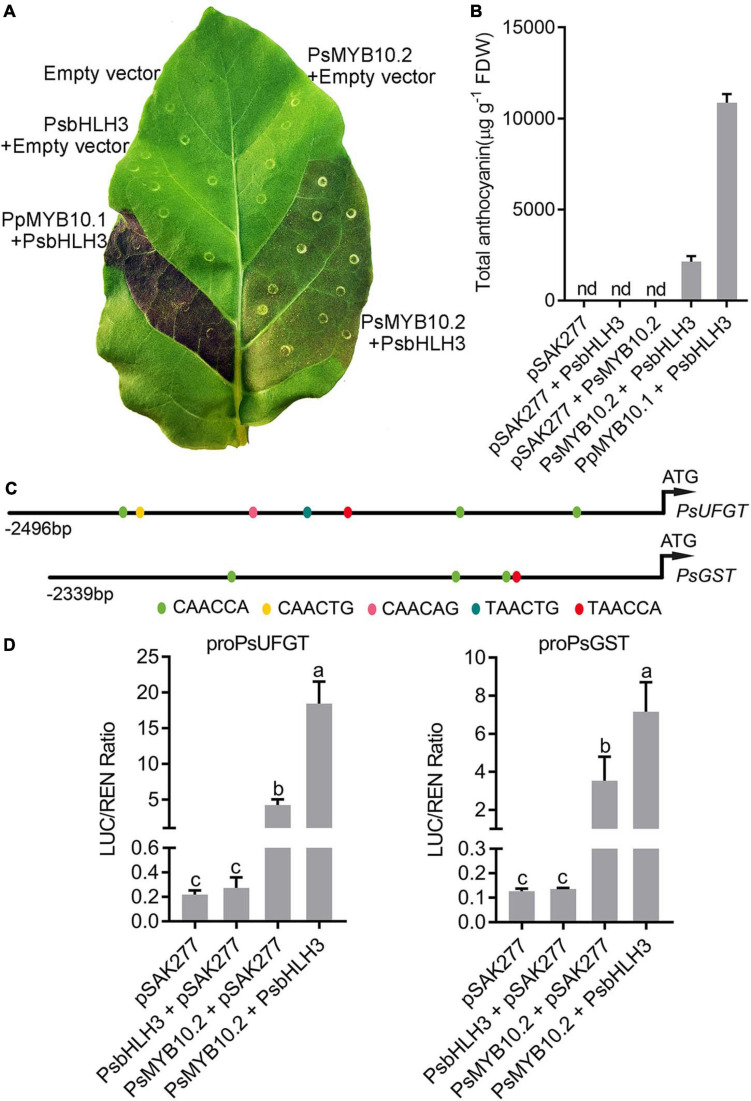
Functional analysis of the *PsMYB10.2* gene using transient assay in tobacco. **(A)** Transient expression of *PsMYB10.2* in tobacco leaf. The photo was taken 7 days after infiltration. Peach *PpMYB10.1*/*PsbHLH3* was used as positive control. **(B)** Anthocyanin content in transformed leaves of tobacco. Each value represents the mean of three biological replicates. FDW, freeze dry weight. nd, non-detected. **(C)** Schematic diagram of the *PsUFGT* and *PsGST* promoter. The prediction of the cis-acting elements in the promoter of *PsUFGT* and *PsGST* was performed using the PlantCARE (http://bioinformatics.psb.ugent.be/webtools/plantcare/html/) database. Color ellipses represent predicted MYB binding sites. **(D)** Analysis of the interaction of the *PsMYB10.2* gene with the promoters of *PsUFGT* and *PsGST* genes using dual-luciferase reporter assay *Nicotiana benthamiana* leaves. Error bars represent the standard errors for three replicates. Different lowercase letters indicate significant differences among treatments according to one-way ANOVA test (*P* < 0.05). Means with the same letter are not significantly different at the 0.05 level.

The cDNA sequence of PsSY0011279 was cloned from the red flesh of ‘Fuhongli’ and designated as *PsMYB10.2* (GenBank accession No. MK340932). The coding sequence of *PsMYB10.2* is identical to the differentially expressed *R2R3 MYB* gene identified from red-fleshed ‘Furongli’ plum in our previous study (Fang et al., 2016b). *PsMYB10.2* was nearly undetectable in the flesh of ‘Sanyueli’ but its expressed increased in the flesh of ‘Fuhongli’ during pigmentation (Figure 4). Tobacco leaf transient expression assays were carried out to test the function of *PsMYB10.2*. Coinfiltration of *PsMYB10.2* and *PsbHLH3* resulted in faint red coloration at infiltration sites 7 days after transformation, but no pigmentation was found when *PsMYB10.2* or *PsbHLH3* were infiltrated alone (Figure 7A). Quantification of anthocyanins in leaves of *N. tabacum* indicated that no anthocyanin was detected in the infiltrated areas transformed with empty vector, *PsMYB10.2* or *PsbHLH3* (Figure 7B). Anthocyanin was detected in tobacco leaves infiltrated with *PsMYB10.2* and *PsbHLH3*, but the content was significantly lower than that in tobacco leaves infiltrated with *PpMYB10.1* and *PsbHLH3* (Figure 7B). In addition, promoter structure analysis revealed there are multiple potential MYB binding sites in the promoter of *PsUFGT* and *PsGST* (Figure 7C). The promoters of *PsUFGT* and *PsGST* were isolated and fused to a luciferase reporter and dual luciferase assays were employed to investigate the interaction of *PsMYB10.2* with the promoter sequences of structural genes involved in anthocyanin accumulation. Infiltration of *PsMYB10.2* was able to activate the promoters of both *PsUFGT* and *PsGST*, and co-infiltration with *PsbHLH3* enhanced the activity of *PsMYB10.2* (Figure 7D). These results confirmed the role of *PsMYB10.2* as a positive regulator of anthocyanin accumulation.

### Silencing of the *PsMYB10.2* Reduces Anthocyanin Accumulation in Flesh of ‘Fuhongli’

**FIGURE 8:**
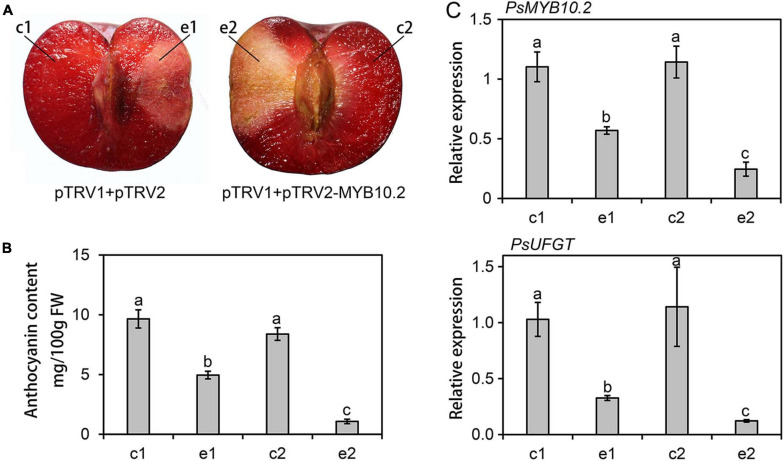
Functional analysis of the *PsMYB10.2* gene by TRV-based virus-induced gene silencing. **(A)** Silencing of the *PsMYB10.2* gene in ‘Fuhongli’ plum fruit at ripening stages. **(B)** Anthocyanin content in the flesh tissues around the infiltration sites (e1 and e2) and the opposite non-infiltrated sites (c1 and c2). FW, fresh weight. **(C)** Expression levels of the *PsMYB10.2* and *PsUFGT* in the flesh tissues. The flesh tissues were collected 7 days after infiltration. Error bars represent the standard errors for three replicates. Different lowercase letters indicate significant differences among treatments according to one-way ANOVA test (*P* < 0.05).

To further validate the role of the *PsMYB10.2*, the VIGS system was used to transiently suppress the expression of *PsMYB10.2* in the flesh of ‘Fuhongli’. Coinjection with pTRV1 and pTRV2-PsMYB10.2 reduced pigmentation in infiltration sites (Figure 8A). The anthocyanin content in the flesh around the injection site of pTRV1/pTRV2-PsMYB10.2 was significantly lower than the corresponding opposite non-injected site (Figure 8B). Anthocyanin content in the flesh tissues around the infiltration site of pTRV1/pTRV2 was reduced, but significantly higher than that of pTRV1/pTRV2-PsMYB10.2. In addition, the expression level of *PsMYB10.2* and *PsUFGT* at the injection site of pTRV1/pTRV2-PsMYB10.2decreased by approximately 57 and 66%, respectively, compared with the injection site of pTRV1/pTRV2 (Figure 8C). These results further confirm that *PsMYB10.2* is involved in the regulation of anthocyanin accumulation in flesh of ‘Fuhongli’.

## Discussion

The association of anthocyanins with fruit quality and potential health benefits, has driven efforts to produce anthocyanin-rich plums through breeding and postharvest treatments in recent years (Cevallos-Casals et al., 2006; Fanning et al., 2014; Santhakumar et al., 2015; Fang et al., 2016a; Wang et al., 2020). To our knowledge, ‘Fuhongli’ is the first red-fleshed mutant induced by γ-radiation. In this study, the mechanism involved in anthocyanin accumulation in the flesh of ‘Fuhongli’ was investigated using HPLC, RNA-Seq, dual-luciferase assays and transient color assays in tobacco leaves and plum fruits.

‘Fuhongli’ is a red-fleshed and late-ripening mutant of ‘Sanyueli’ plum (Fang et al., 2016a). A delayed sugar accumulation was observed in the flesh of ‘Fuhongli’, together with delayed reduction of organic acid concentration through ripening (Figure 1), corroborating its late-ripening trait. Our results indicated also that anthocyanin content increased rapidly in fruit flesh of ‘Fuhongli’ from 115 DAF. The presence of cyanidin-3-rutinoside and cyanidin-3-glucoside, which have been identified as the main anthocyanins in Chinese plum (Fanning et al., 2014), was confirmed in ‘Fuhongli’, while no anthocyanin was detected in flesh of ‘Sanyueli’. Our previous study indicated that the structural genes involved in anthocyanin accumulation were upregulated during fruit ripening (Fang et al., 2016b). It is possible that ‘Fuhongli’ has gained the ability to accumulate anthocyanins due to the activation of anthocyanin biosynthetic genes. In this study, comparison of ‘Sanyueli’ and ‘Fuhongli’ fruit flesh transcriptomes indicated that most genes involved in biosynthesis and accumulation of anthocyanin were also upregulated in flesh of ‘Fuhongli’. On the other hand, most biosynthetic enzymes were lowly expressed or decreased in flesh of ‘Sanyueli’ flesh during ripening. The transcripts of *PsUFGT* and *PsGST* were barely in ‘Sanyueli’ flesh. This suggests a direct link to the lack of anthocyanin accumulation in flesh of ‘Sanyueli’.

The upregulation of structural genes suggested that anthocyanin accumulation in ‘Fuhongli’ flesh might be transcriptionally regulated. Several studies have suggested *R2R3 MYB*s as key genes for anthocyanin accumulation in Rosaceae species (Lin-Wang et al., 2010; Rahim et al., 2014; Gu et al., 2015). Our previous study showed that *PsMYB10.2* was likely the key gene involved in anthocyanin biosynthesis in flesh of ‘Furongli’ plum (Fang et al., 2016b). Results from the RNA-seq and qRT-PCR analyses performed in this study further indicated that transcripts encoding *PsMYB10.2* were significantly upregulated in flesh of ‘Fuhongli’ but not expressed in flesh of ‘Sanyueli’. Sequence analysis indicated that PsMYB10.2 belongs to the anthocyanin activator clade, similar to peach PpMYB10.2 (Zhou et al., 2016, 2018) and shows a high amino acid sequence homology to the cherry plum activator PcMYB10.2. Ectopic expression of *PsMYB10.2* and *PsbHLH3* resulted in anthocyanin accumulation and red pigmentation in tobacco leaves, but no pigmentation was observed when *PsMYB10.2* was infiltrated alone. *PsMYB10.2* silencing by VIGS reduced the expression of *PsUFGT* and the concentration of anthocyanins in the flesh of ‘Fuhongli’. These results suggested that PsMYB10.2 is a key anthocyanin activator responsible for red pigmentation in the flesh of ‘Fuhongli’.

The upregulation of *PsMYB10.2* in flesh of ‘Fuhongli’ suggests mutations are responsible for its transcriptional activation. For example, changes in the upstream regulators of *PsMYB10.2* or a loss of repression. Consistent with this, several transcription factors that were more abundant in the flesh ‘Fuhongli’ or ‘Sanyueli’ were identified. These include the transcription factors that have been suggested to regulate the expression of anthocyanin MYB activators such as NAC (Zhou H. et al., 2015) and MADS (Jaakola et al., 2010). Interestingly, our results demonstrated that the expression level of *PsLBD4* was upregulated and more abundant in the flesh of ‘Fuhongli’. *LBD* genes were first identified for their role in lateral organ development in Arabidopsis (Shuai et al., 2002) and have been reported to be involved in regulation of anthocyanin biosynthesis but they act as repressors. Rubin et al. (2009) demonstrated that AtLBD37, AtLBD38, and AtLBD39 are anthocyanin repressors. They showed that overexpression of these genes inhibited nitrogen deficiency induced anthocyanin via suppressing the expression of anthocyanin activators *PAP1* and *PAP2*, while loss of function enhanced anthocyanin accumulation. Li et al. (2017) reported a nitrate-induced LBD gene *MdLBD13*, a homolog of Arabidopsis LBD37/38, repressed anthocyanin biosynthesis by reducing expression of the flavonoid pathway genes in apple. However, Zhang et al. (2019) found that CsLOB_3 and CsLBD36_2 can bind to and activate the promoter of structural genes involved in flavonoid biosynthesis. However, it remains to be investigated as to whether these transcription factors are involved in regulating the expression of *PsMYB10.2* and anthocyanin biosynthesis in the flesh of ‘Fuhongli’.

The *MYB* responsible for anthocyanin biosynthesis in peach flesh is *PpMYB10.1* (Zhou H. et al., 2015), while *PpMYB10.2* is involved in peach flower coloration (Zhou et al., 2016). Tuan et al. (2015) showed that *PpMYB10.2* was expressed in peach leaves that do not contain anthocyanin. Gu et al. (2015) suggested that *PcMYB10.2* is involved in purple coloration in sepals of cv. Ziyeli. They showed that *PcMYB10.2* was expressed in green-colored flesh of cherry plum, but it was undetectable in purple-colored flesh (Gu et al., 2015). High expression of *PcMYB10.2* was also observed in both purple and green fruit skin, but its expression was much higher in green fruit skin than in purple fruit skin (Gu et al., 2015). These results suggest divergence of the function of *MYB10.2* gene in Rosaceous species.

Our results showed that PsMYB10.2 was closely related to peach PpMYB10.2, the anthocyanin-promoting ability of which is much weaker than PpMYB10.1. Zhou et al. (2018) showed that PpMYB10.2 only induced pale red coloration in tobacco leaves, when infiltrated alone no pigmentation was observed. Similarly, transient expression of *PsMYB10.2* and *PsbHLH3* only induced faint anthocyanin production in tobacco leaves. It is noteworthy that the red coloration induced by *PsMYB10.2* and *PsbHLH3* was observed one a week after infiltration and was stable, while *PpMYB10.2* and *PpbHLH3* induced faint coloration in tobacco leaves 2 weeks after infiltration and the faint pigmentation was only observed about one-third of the replicates (Zhou et al., 2018). Our results demonstrated that *PsMYB10.2* was weaker than *PpMYB10.1* and possibly stronger than *PpMYB10.2*. Sequence alignment indicated that there was an 18-amino acids deletion in C-terminal of *PpMYB10.2.* Whether the 18-amino acids deletion in *PpMYB10.2* is responsible for the difference of anthocyanin-promoting activity between *PsMYB10.2* and *PpMYB10.2* need further investigation. In most cases, the anthocyanin content is low (often <20 mg per 100 g fresh weight) in the flesh of red-fleshed plums and is much lower than that in the peel of red or black skin plums (Fanning et al., 2014; Zhou D. et al., 2015). Similarly low anthocyanin content was also observed in the flesh of ‘Furongli’(Fang et al., 2016b) and ‘Fuhongli’ in this study. As *PsMYB10.2* was responsible for anthocyanin accumulation in the flesh of plum (Fang et al., 2016b), the weak anthocyanin-promoting ability of *PsMYB10.2* may explain the low anthocyanin concentration in flesh of red-fleshed plums. An anthocyanin-rich plum cultivar Queen Garnet, which was shown to contain 107 mg per 100 g fresh weight in flesh, was developed in Queensland (Netzel et al., 2012; Fanning et al., 2014). It would be interesting to identify anthocyanin activators and elucidate underlying mechanisms responsible for anthocyanin biosynthesis in the flesh of anthocyanin-rich plum cultivars such as Queen Garnet.

Our study shows that the direct mechanism for the presence of red color in the flesh of ‘Fuhongli’ plum fruit is via the upregulation of the anthocyanin biosynthetic and transport pathway genes. This is mediated by the transcriptional activator *PsMYB10.2.* Further work will be required to establish the causation of *PsMYB10.2* upregulation in the mutagenized ‘Fuhongli’ plum. Since this is unlikely to be due to actual changes in the coding sequences of the activating MYBs, then it may be due to changes in upstream regulators of the MYB or some loss of repression. Future studies should focus on investigating mutations that are responsible for activating the expression of *PsMYB10.2* in flesh of ‘Fuhongli’. Taken together, the results of our study provide useful information for the development of new red-fleshed plums.

## Data Availability Statement

The datasets presented in this study can be found in online repositories. The names of the repository/repositories and accession number(s) can be found below: https://www.ncbi.nlm.nih.gov/genbank/, PRJNA690967.

## Author Contributions

Z-ZF and X-FY supervised the project. Z-ZF designed and performed the whole experiments and wrote the manuscript. D-RZ, Y-JL, and C-CJ participate in the experiments and data analysis. KL-W and RE provided scientific suggestion and revised the manuscript. SL-P prepared and handled plum samples. CA performed quantification of anthocyanins in tobacco leaves and revised the manuscript. All authors read and approved the final manuscript.

## Conflict of Interest

The authors declare that the research was conducted in the absence of any commercial or financial relationships that could be construed as a potential conflict of interest.

## Footnotes

1 http://www.fao.org/faostat/zh/#data/QC

2 http://planttfdb.cbi.pku.edu.cn/prediction.php

## Supplementary Material

Supplementary Figure 1 Heat map of correlation coefficients between samples.

Supplementary Figure 2 Differentially expressed genes (DEG) identified by RNA-seq analysis in the flesh of ‘Sanyueli’ and the mutant. **(A)** DEGs of different comparison groups. **(B)** Venn diagram showing the number of differentially expressed genes. SY indicates DEGs during ripening of ‘Sanyueli’, MT indicates DEGs during ripening of the red-fleshed mutant, SY vs. MT indicates DEGs between ‘Sanyueli’ and its red-fleshed mutant.

Supplementary Figure 3 Nucleotide alignment of the coding sequence of PsSY0021637, PsSY0023440, PsSY0011279, and PsSY0011777.

Supplementary Figure 4 Gene structure of PsSY0021637, PsSY0023440, PsSY0011279, and PsSY0011777. **(A)** Schematic overview of introns and exons in PsSY0021637, PsSY0023440, PsSY0011279, and PsSY0011777. **(B)** Structural alignments of PsSY0011279 and PsSY0011777. Boxes and lines represent exons and introns, respectively.

Supplementary Table 1 Primer sequences for qRT-PCR analysis.

Supplementary Table 2 Sequences of primers used for vector construction.

Supplementary Table 3 Summary of sequencing and mapping results.

Supplementary Table 4 Summary of functional annotations for putative new genes.

Supplementary Table 5 Genes assigned to phenylpropanoid biosynthesis and flavonoid biosynthesis pathway.

Supplementary Table 6 Differentially expressed transcription factor genes.

## Abbreviations

4CL: 4-coumaroyl:CoA-ligase
ANS: anthocyanin synthase
C4H: cinnamate-4-hydroxylase
CHI: chalcone isomerase
CHS: chalcone synthase
DAF: days after flowering
DFR: dihydroflavonol-4-reductase
DEG: differentially expressed genes
F3H: flavanone-3-hydroxylase
F3′H: flavonoid3′-hydroxylase
FDW: freeze dry weight
FPKM: fragments per kilobase of transcript per million fragments mapped
GO: gene ontology
GST: glutathione S-transferase
HPLC: high performance liquid chromatography
KEGG: Kyoto Encyclopedia of Genes and Genomes
MBW: MYB-bHLH-WD40
NR: non-redundant protein sequence database
PAL: phenylalanine ammonia-lyase
qRT-PCR: real-time quantitative RT-PCR
UFGT: UDPglucose:flavonoid-3-O-glucosyltransferase
VIGS: virus-induced gene silencing
Swiss-Prot: Swiss-Prot protein database
COG: Clusters of Orthologous Groups database
KOG: euKaryotic Ortholog Groups of proteins database
Pfam: protein families database
LBD: LOB domain-containing protein.
