# Supplementary figures and images for: Activation of PsMYB10.2 Transcription Causes Anthocyanin Accumulation in Flesh of the Red-Fleshed Mutant of ‘Sanyueli’ (Prunus salicina Lindl.)
Source: Front Plant Sci. 2021 Jun 22;12:680469. doi: 10.3389/fpls.2021.680469 (PMC8259629; doi:10.3389/fpls.2021.680469)

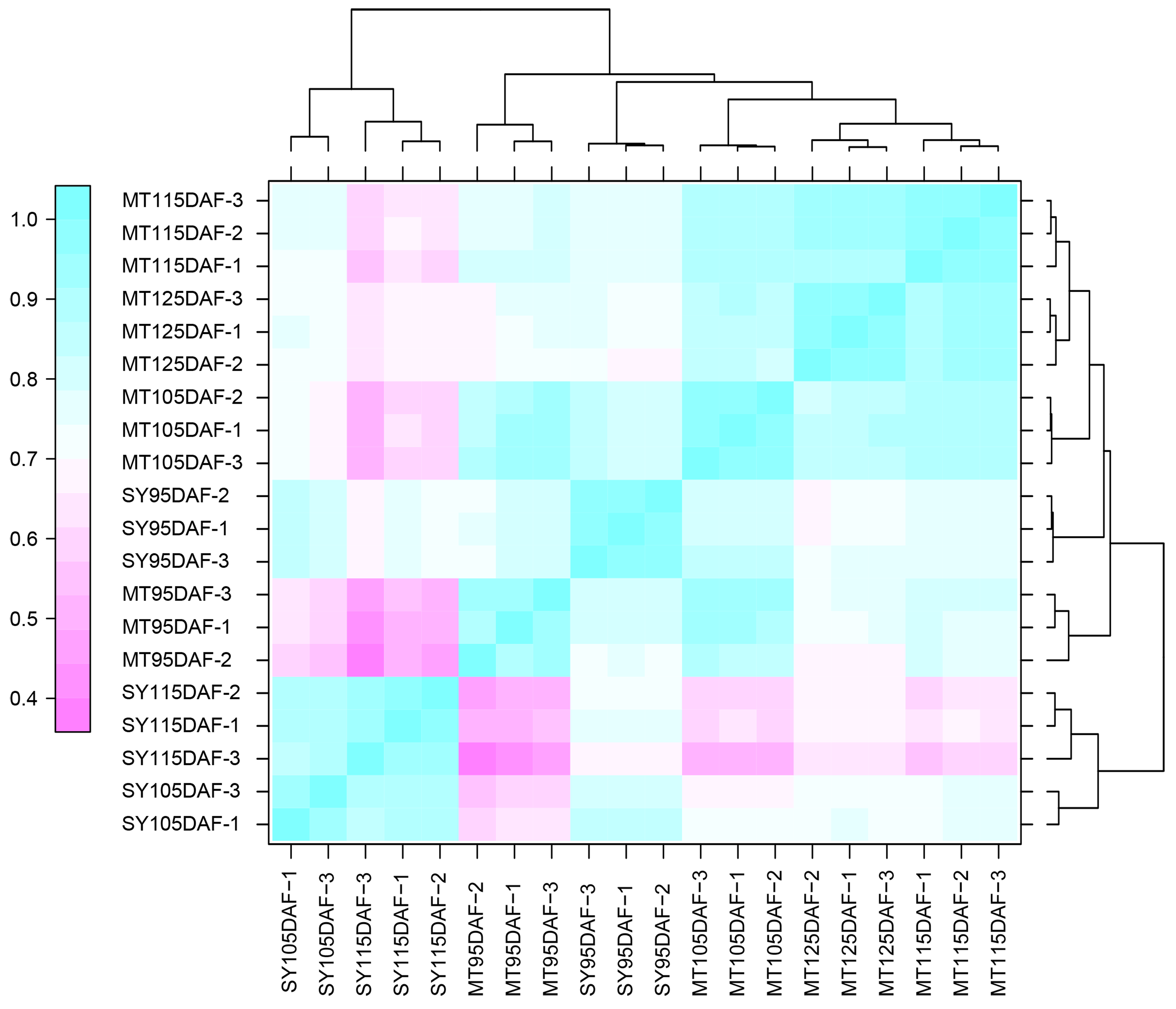

Supplement: Supplementary Figure 1 — Heat map of correlation coefficients between samples. [file Image_1.TIF]

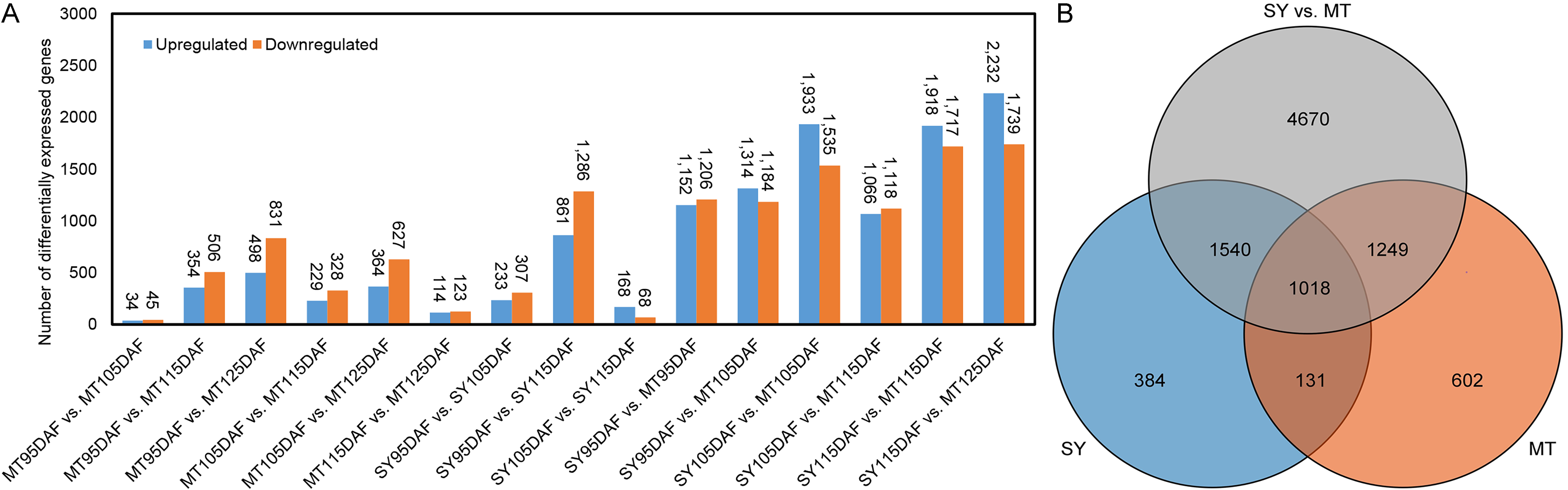

Supplement: Supplementary Figure 2 — Differentially expressed genes (DEG) identified by RNA-seq analysis in the flesh of ‘Sanyueli’ and the mutant. (A) DEGs of different comparison groups. (B) Venn diagram showing the number of differentially expressed genes. SY indicates DEGs during ripening of ‘Sanyueli’, MT indicates DEGs during ripening of the red-fleshed mutant, SY vs. MT indicates DEGs between ‘Sanyueli’ and its red-fleshed mutant. [file Image_2.TIF]

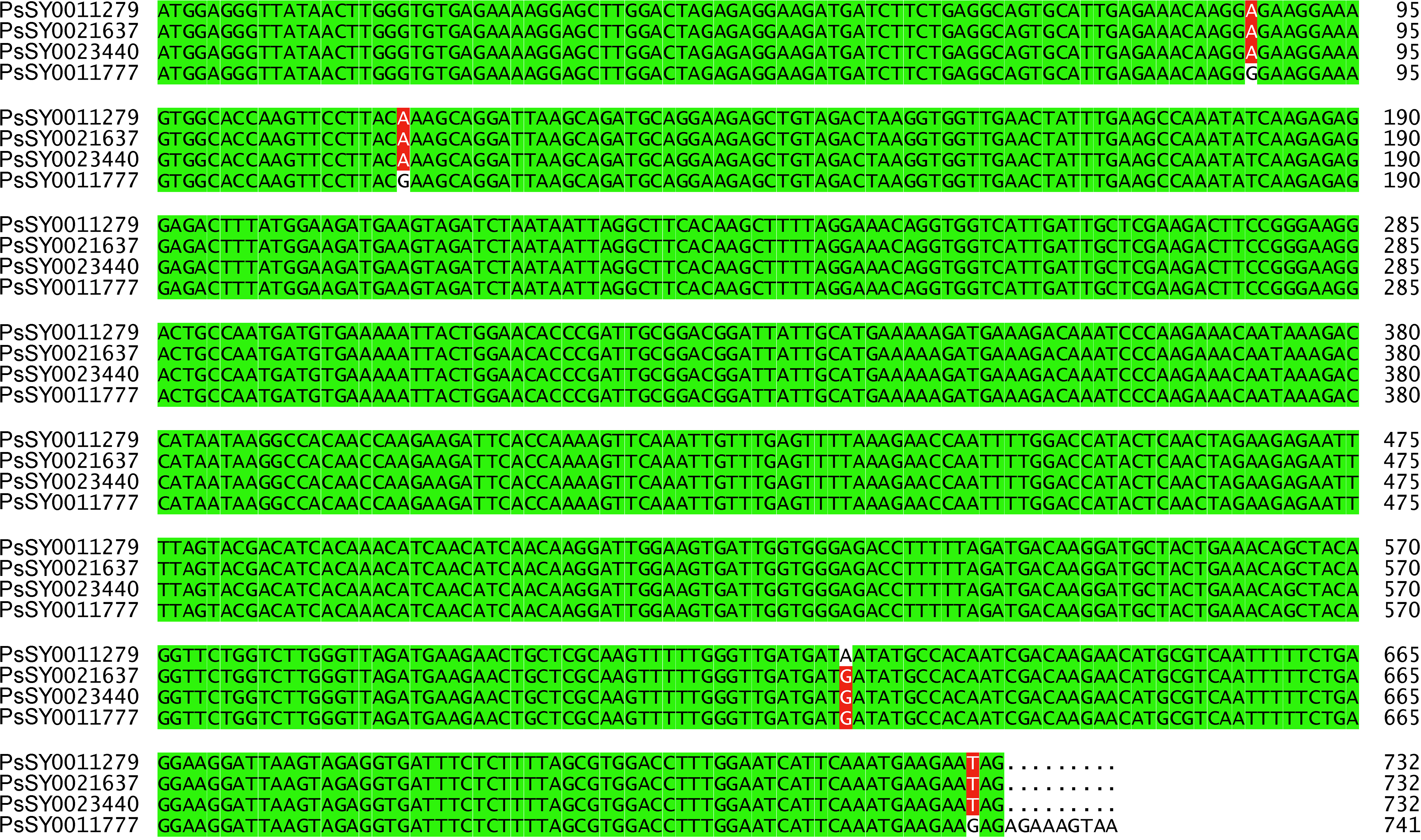

Supplement: Supplementary Figure 3 — Nucleotide alignment of the coding sequence of PsSY0021637, PsSY0023440, PsSY0011279, and PsSY0011777. [file Image_3.TIF]

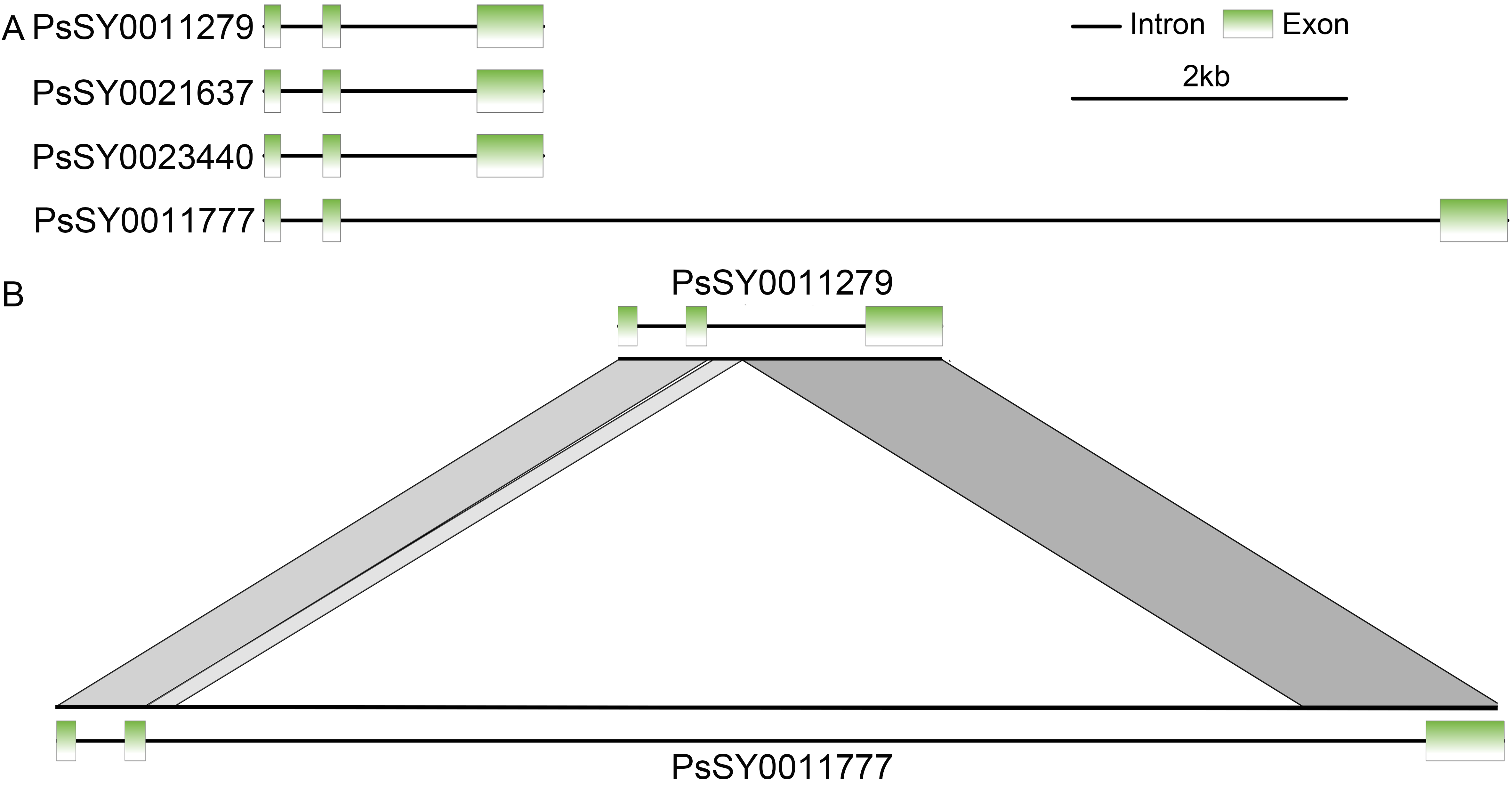

Supplement: Supplementary Figure 4 — Gene structure of PsSY0021637, PsSY0023440, PsSY0011279, and PsSY0011777. (A) Schematic overview of introns and exons in PsSY0021637, PsSY0023440, PsSY0011279, and PsSY0011777. (B) Structural alignments of PsSY0011279 and PsSY0011777. Boxes and lines represent exons and introns, respectively. [file Image_4.TIF]
