# Supplementary material for: Activation of PsMYB10.2 Transcription Causes Anthocyanin Accumulation in Flesh of the Red-Fleshed Mutant of ‘Sanyueli’ (Prunus salicina Lindl.)
Source: Front Plant Sci. 2021 Jun 22;12:680469. doi: 10.3389/fpls.2021.680469 (PMC8259629; doi:10.3389/fpls.2021.680469)
Supplement: Supplementary Table 1 — Primer sequences for qRT-PCR analysis. [file Table_1.doc]

**[Supplementary](https://www.frontiersin.org/articles/10.3389/fpls.2021.624319/full#S8) Table S1** Primer sequences for qRT-PCR analysis.

| Genes | Unigene ID | Forward primer | Reverse primer |
| --- | --- | --- | --- |
| *PAL* | PsSY0020810 | ACATGGCAACCAACTCCATCAAGC | GCTCAACCTTGACCCCAGAGTCATG |
| *C4H* | PsSY0029112 | GACGCTCAGCAGAAGGGAGAGATC | GCACTGAGTCAAGCTCATCCCTCAG |
| *4CL* | PsSY0016602 | GTACAGGGTCAGCTACATGCC | CCGTATCCCTGCACAAGTTCC |
| *CHS* | PsSY0019243 | ACGGAAACATGTCCAGTGCC | GGTCTCAACAGTGAGTCCTGGT |
| *CHI* | PsSY0021513 | GTGACTGTTGTGGGACCTACGATC | GAAGTGTCCTTGTGTGCCATGAGAG |
| *F3H* | PsSY0015860 | CCACATTCCAGAACCCAGCT | TCCTGCGATTGTTGCTCCTT |
| *F3’H* | PsSY0012653 | AAGACAGTGAACTTGGCTCAGC | CTCCTGCCAACACCATCATCTC |
|  | PsSY0020267 | GGCAGGTGGTGAAGACTTCG | ATTGCCCATTCGACGGTCAC |
| *DFR* | PsSY0000799 | GCGATGTTGAATTTTGCCGC | AAGTCCGGTGATGAGGCTTG |
| *ANS* | PsSY0019761 | GAGAGAGATGCAGGGAGGAGTTG | CTGGTCATTGGCGTACATCTCC |
| *UFGT* | PsSY0022880 | GTGGAGGACGTGTTGGACAT | CCGACTGTTGTGCGAGTAGT |
| *GST* | PsSY0017871 | GGTACTTCAACTTCTGGTGCTGC | CCTGGAAGATGGCTCAGATCAG |
| *MYB10.2* | PsSY0011279 | CAAGGAGAAGGAAAGTGGCACC | CCCGGAAGTCTTCGAGCAATC |
| *ICE1* | PsSY0029118 | CTGAACGGTGCCGTTTGGAT | GGGCAGATACCAATCATCAACATC |
| *LBD4* | PsSY0017735 | GATGCCCTCCAAACCCAATTG | TGGCCTGATCAACCACCATGTC |
| *YABBY4* | PsSY0006894 | GCTTCCAGTGAACATGCGTG | GGGGCCTTGGAAGTTCATCA |
| *ACTIN* | PsSY0026636 | TGCTGGACTCTGGTGATGGTG | ACAATTTCCCGCTCAGCAGTG |
