# Supplementary material for: Activation of PsMYB10.2 Transcription Causes Anthocyanin Accumulation in Flesh of the Red-Fleshed Mutant of ‘Sanyueli’ (Prunus salicina Lindl.)
Source: Front Plant Sci. 2021 Jun 22;12:680469. doi: 10.3389/fpls.2021.680469 (PMC8259629; doi:10.3389/fpls.2021.680469)
Supplement: Supplementary Table 2 — Sequences of primers used for vector construction. [file Table_2.docx]

[**Supplementary**](https://www.frontiersin.org/articles/10.3389/fpls.2021.624319/full#S8) **Table S2 Sequences of primers used for vector construction**

| Primer name | Sequence | Description |
| --- | --- | --- |
| PsMYB10.2OEF | actagtggatccaaagaattcATGGAGGGTTATAACTTGGGTGTG | Primers used for inserting *PsMYB10.2* into vector pSAK277 |
| PsMYB10.2OER | agaagtactctcgagaagcttCTATTCTTCATTTGAATGATTCCAAAG |  |
| PsbHLH3OEF | actagtggatccaaagaattcATGATGGCTGCACCGCCA | Primers used for inserting *PsbHLH3* into vector pSAK277 |
| PsbHLH3OER | agaagtactctcgagaagcttCTAGGAATCAGATTGGGGAATTATTT |  |
| pTRV2-PsMYB10.2F | taaggttaccgaattcATGTGAAAAATTACTGGAACACCCG | Primers used for gernerating pTRV2 Vector of PsMYB10.2 |
| pTRV2-PsMYB10.2R | agacgcgtgagctcgTCTTGTCGATTGTGGCATATTATC |  |
| proUFGT-LUCF | gtcgacggtatcgataagcttGGCCACCAACATCCACATTC | Primers used for inserting proPsUFGT into vector pGreenII LUC+ |
| proUFGT-LUCR | agtggatcccccgggctgcagATATGTATGAGGTAATAAGACTAACAGGAG |  |
| proGST-LUCF | gtcgacggtatcgataagcttCAGGCACTTGCGAATAGAGCA | Primers used for inserting proPsGST into vector pGreenII LUC+ |
| proGST-LUCR | agtggatcccccgggctgcagCTTGTTATAGTAATATCTCTCTCACTCCTT |  |
