# Supplementary material for: Activation of PsMYB10.2 Transcription Causes Anthocyanin Accumulation in Flesh of the Red-Fleshed Mutant of ‘Sanyueli’ (Prunus salicina Lindl.)
Source: Front Plant Sci. 2021 Jun 22;12:680469. doi: 10.3389/fpls.2021.680469 (PMC8259629; doi:10.3389/fpls.2021.680469)
Supplement: Supplementary Table 3 — Summary of sequencing and mapping results. [file Table_3.docx]

[**Supplementary**](https://www.frontiersin.org/articles/10.3389/fpls.2021.624319/full#S8) **Table S3 Summary of sequencing and mapping results**

| Sample Name | Clean Reads | Clean bases (bp) | GC Content | %≥Q30 | Map Rate |
| --- | --- | --- | --- | --- | --- |
| SY95DAF-1 | 54,873,892 | 9,631,564,764 | 48.24% | 93.23% | 85.09% |
| SY95DAF-2 | 49,912,166 | 8,048,643,378 | 48.34% | 92.98% | 84.07% |
| SY95DAF-3 | 66,075,800 | 7,341,899,692 | 48.12% | 92.80% | 83.93% |
| SY105DAF-1 | 57,937,222 | 9,735,848,796 | 48.03% | 92.44% | 84.30% |
| SY105DAF-2 | 60,048,762 | 8,533,044,408 | 48.29% | 91.97% | 83.85% |
| SY105DAF-3 | 55,792,550 | 8,836,365,458 | 48.22% | 92.23% | 84.81% |
| SY115DAF-1 | 40,997,452 | 8,206,723,306 | 48.16% | 92.31% | 84.78% |
| SY115DAF-2 | 44,948,548 | 6,009,916,968 | 47.94% | 92.21% | 85.35% |
| SY115DAF-3 | 42,796,408 | 6,565,345,202 | 48.04% | 91.74% | 81.38% |
| MT95DAF-1 | 44,921,200 | 6,586,104,040 | 48.12% | 93.08% | 82.76% |
| MT95DAF-2 | 44,766,314 | 6,592,379,038 | 48.36% | 93.54% | 83.29% |
| MT95DAF-3 | 45,128,100 | 6,651,242,124 | 48.18% | 92.94% | 82.22% |
| MT105DAF-1 | 52,812,564 | 7,775,183,456 | 47.85% | 92.08% | 81.56% |
| MT105DAF-2 | 50,819,566 | 7,485,910,158 | 47.91% | 92.08% | 81.65% |
| MT105DAF-3 | 48,119,708 | 7,076,917,984 | 47.79% | 92.19% | 84.57% |
| MT115DAF-1 | 46,639,126 | 6,835,874,942 | 48.05% | 92.41% | 81.71% |
| MT115DAF-2 | 46,048,362 | 6,766,511,730 | 48.04% | 92.39% | 81.45% |
| MT115DAF-3 | 60,628,602 | 8,945,090,920 | 47.81% | 92.05% | 81.82% |
| MT125DAF-1 | 69,757,140 | 10,291,635,882 | 47.81% | 91.87% | 81.29% |
| MT125DAF-2 | 61,566,842 | 9,078,455,202 | 47.75% | 92.23% | 82.02% |
| MT125DAF-3 | 65,170,374 | 6,586,104,040 | 47.41% | 91.99% | 82.30% |
