# Supplementary material for: Activation of PsMYB10.2 Transcription Causes Anthocyanin Accumulation in Flesh of the Red-Fleshed Mutant of ‘Sanyueli’ (Prunus salicina Lindl.)
Source: Front Plant Sci. 2021 Jun 22;12:680469. doi: 10.3389/fpls.2021.680469 (PMC8259629; doi:10.3389/fpls.2021.680469)
Supplement: Supplementary Table 4 — Summary of functional annotations for putative new genes. [file Table_4.docx]

[**Supplementary**](https://www.frontiersin.org/articles/10.3389/fpls.2021.624319/full#S8) **Table S4 Summary of functional annotations for putative new genes**

| Annotated databases | New Gene Number |
| --- | --- |
| GO | 652 |
| KEGG | 438 |
| KOG | 667 |
| Pfam | 617 |
| Swiss-Prot | 722 |
| eggNOG | 1,048 |
| nr | 1,630 |
| All | 1,641 |
